# Verteporfin reverses progestin resistance through YAP/TAZ-PI3K-Akt pathway in endometrial carcinoma

**DOI:** 10.1038/s41420-023-01319-y

**Published:** 2023-01-25

**Authors:** Lina Wei, Xiaohong Ma, Yixin Hou, Tianyi Zhao, Rui Sun, Chunping Qiu, Yao Liu, Ziyi Qiu, Zhiming Liu, Jie Jiang

**Affiliations:** 1 Department of Gynecology and Obstetrics, Qilu Hospital of Shandong University, 107 Wenhua Xi Road, 250012 Jinan, Shandong China; 2 Gynecology Oncology Key Laboratory, Qilu Hospital, Shandong University, Jinan, Shandong China; 3 Department of Gynecology and Obstetrics, the Affiliated Yantai Yuhuangding Hospital of Qingdao University, 20 Yuhuangding East Road, 264000 Yantai, Shandong China

**Keywords:** Cancer therapeutic resistance, Cancer prevention

## Abstract

Progestin resistance is a problem for patients with endometrial carcinoma (EC) who require conservative treatment with progestin, and its underlying mechanisms remain unclear. YAP and TAZ (YAP/TAZ), downstream transcription coactivators of Hippo pathway, promote viability, metastasis and also drug resistance of malignant tumors. According to our microarray analysis, YAP/TAZ were upregulated in progestin resistant IshikawaPR cell versus progestin sensitive Ishikawa cell, which implied that YAP/TAZ may be a vital promotor of resistance to progestin. We found YAP/TAZ had higher expression levels among the resistant tissues than sensitive tissues. In addition, knocking down YAP/TAZ decreased cell viability, inhibited cell migration and invasion and increased the sensitivity of IshikawaPR cell to progestin. On the contrary, overexpression of YAP/TAZ increased cell proliferation, metastasis and promoted progestin resistance. We also confirmed YAP/TAZ were involved in progestin resistant process by regulating PI3K-Akt pathway. Furthermore, Verteporfin as an inhibitor of YAP/TAZ could increase sensitivity of IshikawaPR cells to progestin in vivo and in vitro. Our study for the first time indicated that YAP/TAZ play an important role in progestin resistance by regulating PI3K-Akt pathway in EC, which may provide ideas for clinical targeted therapy of progestin resistance.

## Introduction

Endometrial carcinoma (EC) is rising in incidence and mortality worldwide and the age of onset tends to be younger [1]. Although surgery is the standard and basic treatment for most EC patients, continuous progestin-based treatment, including oral medroxyprogesterone acetate (MPA), is the common option for the women of childbearing age who want to preserve reproductive function [2–5]. However, clinical studies [3, 5, 6] reported that 48.2% to 55% of the patients with stage IA, grade 1 endometrioid adenocarcinoma completely respond to conservative treatment including MPA, however, the recurrence rate in these patients is up to 33.8%. There are other studies [7, 8] showed that more than 30% of EC patients initially fail to respond to progestin and develop progestin resistance during the course of progestin treatment. Therefore, progestin resistance is a huge obstacle to undergoing medical treatment and its potential mechanisms remain unclear.

YAP/TAZ are transcriptional coactivators and commonly known as the downstream effectors of Hippo pathway which was originally discovered in Drosophila [9]. In a variety of tumors such as breast, lung, colorectal, and intestinal cancers, YAP and TAZ play distinct and overlapping functions as they are regulated by multiple factors [10]. In breast cancer, for example, both YAP and TAZ are highly expressed in a variety of breast tumor subtypes; however, YAP can function as oncogene or proto-oncogene due to the different breast cancer subtypes and the influence of environmental and experimental conditions. In invasive lobular breast cancer, YAP plays a carcinogenic role and high YAP expression and nuclear localization are associated with tumor metastatic ability [11]. The high transcription and stable protein properties of TAZ in breast cancer are effective markers of tumor aggressiveness and poor prognosis [12, 13]. Meanwhile, it has also been shown that YAP and TAZ play similar and necessary roles in regulating tumor growth, and in some cases activation of YAP or TAZ alone is not sufficient to drive tumorigenesis; therefore, therapeutic targeting of both YAP and TAZ can sometimes suppress tumor properties [10, 14–16]. Studies [17–19] have also shown that elevated expression of YAP/TAZ in the nucleus is relevant to malignancy degrees and poor prognosis. YAP/TAZ are regulated by a canonical kinase cascade of Hippo pathway whose upstream regulators include MST1/2 and LATS1/2 [20]. When Hippo pathway is on, cascade phosphorylation of MST1/2 and LATS1/2 results in phosphorylating YAP/TAZ, inducing cytosolic retention and subsequent degradation of YAP/TAZ. Conversely, when Hippo pathway is off, dephosphorylation of MST1/2 and LATS1/2 leads to a decrease in phosphorylated YAP and activated YAP/TAZ will transfer into the nucleus to perform biological functions [21, 22]. Additionally, the activity of YAP/TAZ is regulated directly or indirectly by mechanotransduction, metabolic pathways and other pathways [19, 23, 24]. As regulated by various pathways, YAP/TAZ have been also explored to explain the mechanisms in resistance to cancer therapies including targeted therapies, radiation and chemotherapy [24]. In breast cancer [25], especially, knocking down YAP and TAZ increases sensitivity to tamoxifen in MCF7 cells, which suggests that there might be a correlation between YAP/TAZ and hormone receptor signaling. Hormone therapy including progestin therapy is also one of the treatment options for EC. Evidence suggests that YAP/TAZ are critical for tumorigenesis, tumor growth and metastasis in EC [26–28]. However, the role of YAP/TAZ in progestin resistance of EC is not understood.

Verteporfin is a small molecular photosensitizer which is used to treat neovascular macular degeneration [29]. It has been reported to inhibit proliferation of endometrioid adenocarcinoma cell lines by suppressing YAP/TAZ levels [28]. There are few studies showed that Verteporfin could be used clinically to reverse progestin resistance by targeting YAP/TAZ in EC.

In this study, we hypothesized that YAP/TAZ as coactivators are involved in progestin resistance of EC. We performed experiments in vivo and in vitro to explore the functions and the underlying mechanisms of YAP/TAZ in progestin resistance of EC. It may provide a new therapeutic strategy for progestin resistant therapeutics of endometrial carcinoma.

## Results

### YAP/TAZ are upregulated and activated in IshikawaPR cells

**Fig. 1:**
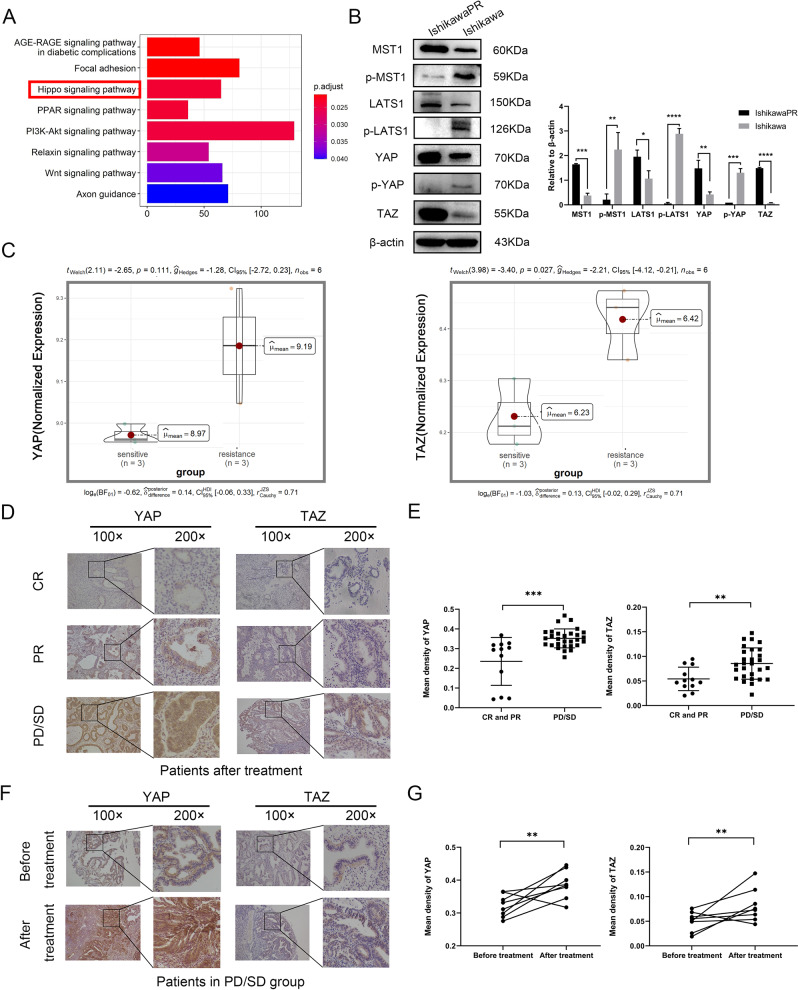
YAP/TAZ are higher expressed in IshikawaPR cells and progestin resistant tissues. **A** The top pathways of KEGG enrichment analysis between Ishikawa cell and IshikawaPR cell. **B** The expression of key molecules of Hippo pathway in IshikawaPR cell and Ishikawa cell confirmed by western blotting assay. **C** The violin plots showing the expression distributions of YAP and TAZ between Ishikawa cell and IshikawaPR cell. **D** Representative images of IHC staining of YAP and TAZ in endometrial tissues after progestin treatment in CR, PR, PD/SD groups. **E** Mean density of YAP and TAZ in endometrium after progestin treatment in CR and PR, PD/SD groups analyzed by Image-Pro Plus 6.0. **F** Representative images of IHC staining of YAP and TAZ in endometrial tissues of each patient before and after progestin treatment in PD/SD group. **G** Mean density of YAP and TAZ in endometrium of each patient before and after progestin treatment in PD/SD group analyzed by Image-Pro Plus 6.0. CR complete response group, PR partial response group, PD/SD progressive disease/stable disease group. *P < 0.05, **P < 0.01, ***P < 0.001, and ****P < 0.0001. Statistical analysis of IHC was performed using Mann–Whitney test and Student’s *t* test.

Microarray analysis was performed on progestin sensitive Ishikawa cell and progestin resistant IshikawaPR cell. Based on the raw data from GEO database (GSE121367), we carried out KEGG enrichment analysis and showed top eight signaling pathways including Hippo pathway (Fig. 1A). We detected the expression levels of key molecules and their phosphorylated forms in the Hippo pathway by western blotting assay. Results showed that in IshikawaPR cells, the phosphorylation of MST1(p-MST1), the phosphorylation of LATS1(p-LATS1) and the phosphorylation of YAP(p-YAP) decreased while YAP and TAZ were upregulated, which means Hippo pathway was off and YAP/TAZ were activated (Fig. 1B, Original Data Fig. 1B). We also performed a western blotting experiment in the nucleus and cytosol fractions of the two cell lines. Results indicated that in IshikawaPR cells the expression of YAP and TAZ was both higher in the nucleus (Supplementary Fig. 1A, Original Data Supplementary Fig. 1A). In IshikawaPR cells the target genes of YAP/TAZ, CTGF, CYR61, and ANKRD1 [30] had higher transcription levels than in Ishikawa cells (Supplementary Fig. 1B). The violin plots revealed that mean YAP mRNA expression was quantified at 9.19 in IshikawaPR cells which was slightly higher than that of Ishikawa cells and TAZ mRNA expressed at higher levels in IshikawaPR cells with *P* < 0.05 (Fig. 1C).

### YAP/TAZ are highly expressed in progestin resistant tissues

**Fig. 2:**
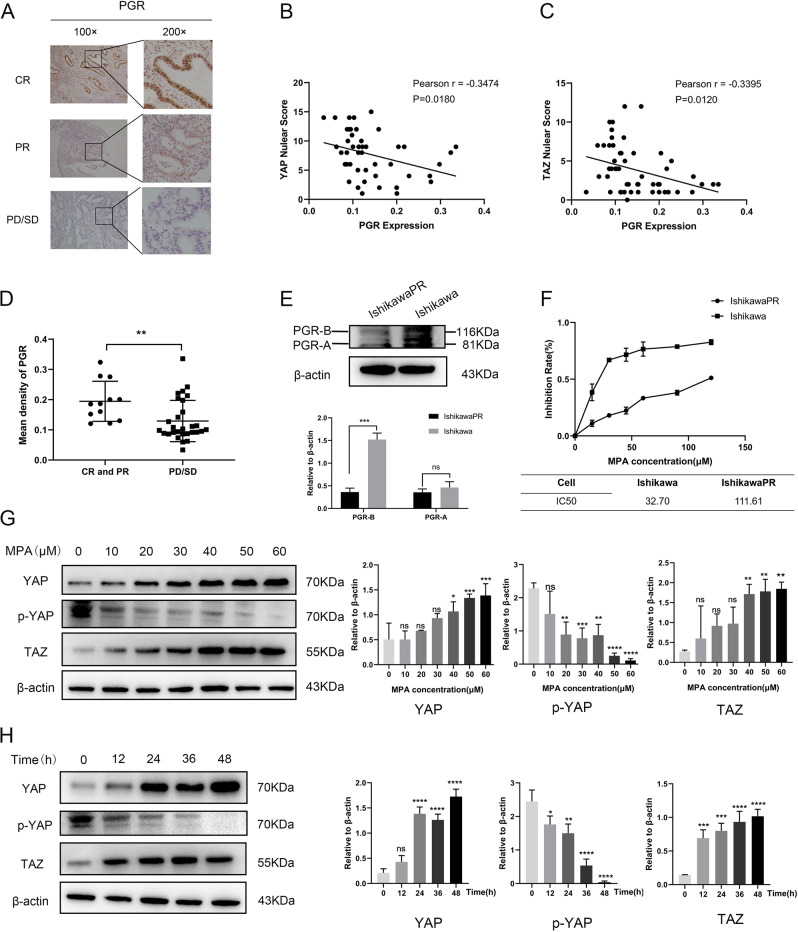
Negative correlations between YAP/TAZ and PGR and the influence of MPA on YAP/TAZ in progestin resistant cells. **A** Representative images of IHC staining of PGR in endometrial tissues after progestin treatment in CR, PR, PD/SD groups. **B** The correlation between the expression of nuclear YAP and the expression of PGR in patient tissues that received progestin treatment. **C** The correlation between the expression of nuclear TAZ and the expression of PGR in patient tissues that received progestin treatment. **D** Mean density of PGR in endometrium after progestin treatment in CR and PR, PD/SD groups analyzed by Image-Pro Plus 6.0. **E** The expression of PGR-A and PGR-B in Ishikawa cell and IshikawaPR cell confirmed by western blotting. **F** The growth curves and IC50 of Ishikawa cell and IshikawaPR cell with different concentrations of MPA. **G** The expression of YAP, p-YAP, and TAZ in IshikawaPR cell with different concentrations of MPA by western blotting. **H** The expression of YAP, p-YAP, and TAZ in IshikawaPR cell at different time points with fixed concentration of MPA by western blotting. *P < 0.05, **P < 0.01, ***P < 0.001, and ****P < 0.0001.

The collected tissues of patients who had regularly received progestin therapy were divided into complete response group (CR), partial response group (PR), progressive disease/stable disease group (PD/SD). Immunohistochemistry (IHC) showed that the expression levels of YAP and TAZ were obviously lower in CR and PR patients than that in PD/SD patients after regular progestin treatment (Fig. 1D, E). Additionally, YAP and TAZ were highly upregulated after treatment compared with before treatment in PD/SD patients (Fig. 1F, G). Progestin receptor (PGR) is a prerequisite for progestin treatment and progestin inhibits cell growth and invasiveness probably mainly through PGR [31]. So, we also detected PGR in clinical tissues by IHC and analyzed the relationship between PGR expression and nuclear YAP and TAZ. We found that the expression of nuclear YAP and TAZ was negatively related to PGR (Fig. 2A–D), which was consistent with results obtained by western blotting assay (Fig. 2E, Original Data Fig. 2E).

### The influence of progestin on YAP/TAZ in IshikawaPR cells

Ishikawa cells and IshikawaPR cells were cultured with different concentrations of MPA for 48 h and proliferation was detected by MTT assay. As the curve shown, the inhibition of MPA on cell viability was more and more obvious with the increase of MPA concentrations. The IC50 values were respectively 32.70 μM of Ishikawa cells and 111.61 μM of IshikawaPR cells (Fig. 2F). Above results indicated that progestin resistant cell was reliable. To investigate the change of YAP, p-YAP and TAZ in response to MPA, western blotting assay was performed. Results showed that high dose MPA treatment caused an increase in YAP and TAZ in IshikawaPR cells. With prolongation of time, YAP and TAZ also increased in IshikawaPR cells. The expression of p-YAP tended to decline in dose-dependent or time-dependent way in IshikawaPR cells (Fig. 2G, H, Original Data Fig. 2G, H). TAZ activity is primarily regulated by protein abundance while YAP activity is by the nuclear-cytoplasmic shuttle [10]. So we analyzed the nuclear and cytoplasmic localization of YAP in the same experimental conditions, and results revealed that with MPA treatment in dose and time dependent ways, YAP might play a role in promoting progestin resistance mainly in the nucleus (Supplementary Fig. 1C, D, Original Data Supplementary Fig. 1C, D). Taken the above results together, we preliminarily made a conclusion that YAP and TAZ were associated with progestin resistance in EC.

### Knocking down YAP/TAZ increases the sensitivity of IshikawaPR cells to MPA

**Fig. 3:**
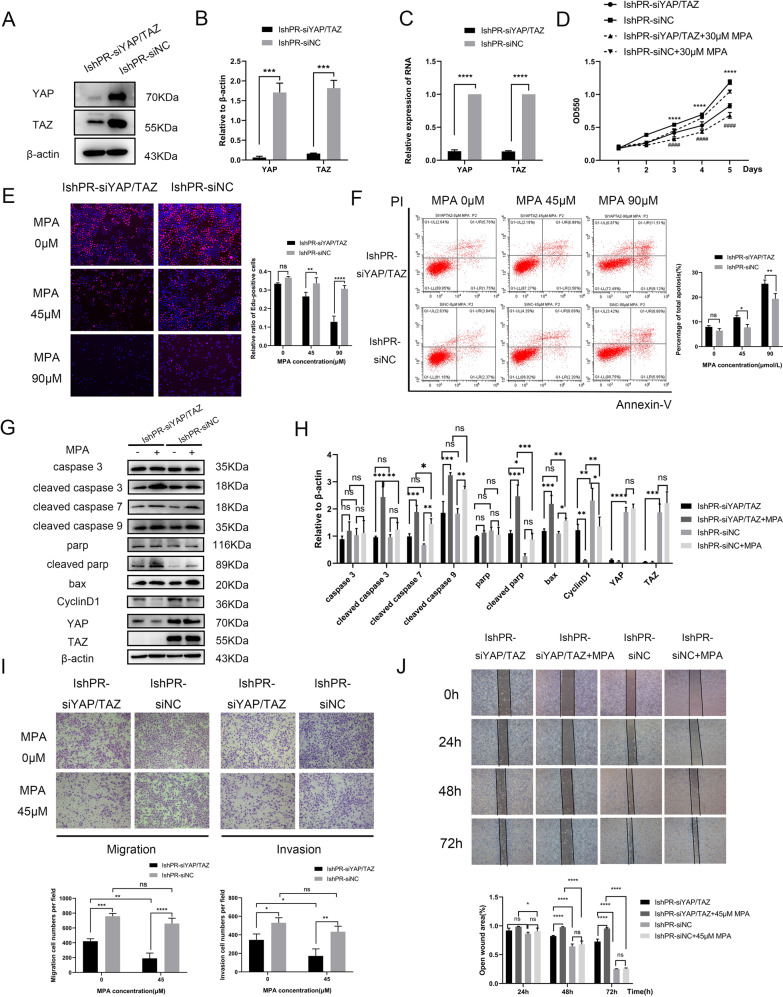
Knocking down YAP/TAZ increases the sensitivity of IshikawaPR cells to MPA. **A** The protein levels of YAP/TAZ in IshPR-siYAP/TAZ and IshPR-siNC cells by western blotting. **B** Statistical graph of expression levels of YAP/TAZ in IshPR-siYAP/TAZ and IshPR-siNC cells in (**A**). **C** The mRNA levels of YAP/TAZ in IshPR-siYAP/TAZ and IshPR-siNC cells by RT-PCR. **D** The growth curves of IshPR-siYAP/TAZ and IshPR-siNC cells at 0, 30 μM MPA examined by MTT assay at 1–5 days. ****P < 0.0001 IshPR-siYAP/TAZ cells without MPA vs IshPR-siNC cells without MPA at the same day. ####*P* < 0.0001 IshPR-siYAP/TAZ cells with MPA vs IshPR-siNC cells with MPA at the same day. **E** EDU incorporation assay after treatment with 0, 45, 90 μM MPA separately for 48 h. **F** The apoptosis of IshPR-siYAP/TAZ and IshPR-siNC cells after treatment with 0, 45, 90 μM MPA separately for 48 h showed by flow cytometry assay. **G** The expression of apoptosis and proliferation protein markers in IshPR-siYAP/TAZ and IshPR-siNC cells after treatment with 0, 45 μM MPA separately. **H** Statistical graph of (**G**). **I** The effects of knocking down YAP/TAZ on migration and invasion capacity of IshikawaPR cells with different concentrations of MPA showed by Transwell assay. **J** The effects of knocking down YAP/TAZ on migration capacity of IshikawaPR cells with different concentrations of MPA showed by wound healing assay. *P < 0.05, **P < 0.01, ***P < 0.001, ****P < 0.0001 and ####*P* < 0.0001.

We knocked down YAP and TAZ alone and together by transfecting small interfering RNA into IshikawaPR cells and the efficiency was confirmed by western blotting and RT-PCR (Fig. 3A–C, Supplementary Fig. 2A, Original Data Fig. 3A, Original Data Supplementary Fig. 2A). IshPR-siYAP and IshPR-siTAZ individually decreased the cell proliferation ability and induced cell apoptosis, whereas knocking down YAP and TAZ (IshPR-siYAP/TAZ) had more remarkable effects especially at the presence of MPA (Supplementary Fig. 2B–D). Therefore, we regarded YAP and TAZ as a critical complex for subsequent experiments. MTT assay displayed that interference of YAP/TAZ decreased proliferation ability of IshikawaPR cells especially at 3–5 days with 0 or 30 μM MPA (Fig. 3D). It also showed that with elevated concentrations of MPA, DNA synthesis of IshPR-siYAP/TAZ cells sharply reduced comparing with IshPR-siNC cells (Fig. 3E, Supplementary Fig. 2E). Furthermore, flow cytometry showed that the percentage of total apoptosis of IshPR-siYAP/TAZ cells was significantly higher than IshPR-siNC cells with MPA administration (Fig. 3F, Supplementary Fig. 2F). Meanwhile, the expression of proliferation and apoptosis proteins was detected after MPA treatment by western blotting. Cleaved caspase 3, cleaved caspase 7, cleaved caspase 9, bax and cleaved parp were higher expressed in IshPR-siYAP/TAZ cells than in IshPR-siNC especially at the presence of MPA, while CyclinD1 was less in IshPR-siYAP/TAZ cells, which was in accord with the results of flow cytometry (Fig. 3G, H, Original Data Fig. 3G). Besides, we also found that silencing YAP/TAZ in IshikawaPR cells more significantly impaired the ability of migration and invasion by Transwell and wound healing assays at different concentrations of MPA (Fig. 3I, J).

### Overexpression of YAP/TAZ decreases the sensitivity of Ishikawa cells to MPA

**Fig. 4:**
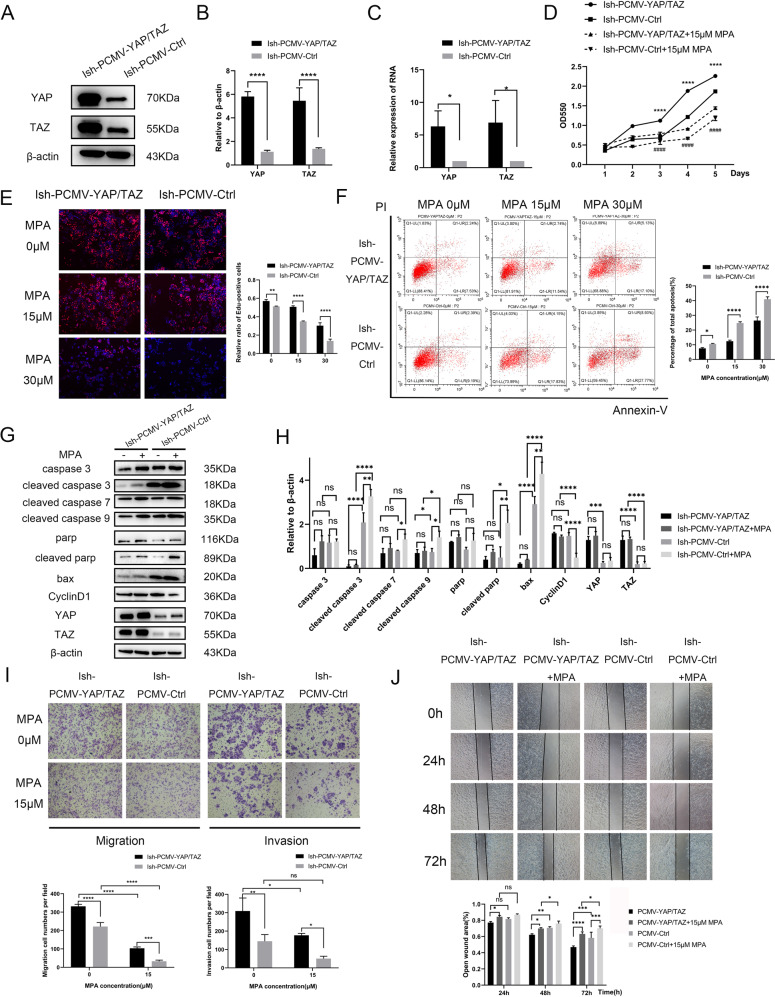
Overexpression of YAP/TAZ decreases the sensitivity of Ishikawa cells to MPA. **A** The efficiency of overexpression of YAP/TAZ in Ishikawa cells detected by western blotting. **B** Statistical graph of overexpression efficiency of YAP/TAZ in Ishikawa cells in (**A**). **C** The efficiency of overexpression of YAP/TAZ in Ishikawa cells detected by RT-PCR. **D** The growth curves of Ish-PCMV-YAP/TAZ and Ish-PCMV-Ctrl cells at 0, 15 μM MPA examined by MTT assay at 1–5 days. ****P < 0.0001 Ish-PCMV-YAP/TAZ cells without MPA vs Ish-PCMV-Ctrl cells without MPA at the same day. ####*P* < 0.0001 Ish-PCMV-YAP/TAZ cells with MPA vs Ish-PCMV-Ctrl cells with MPA at the same day. **E** EDU incorporation assay treated with 0, 15, 30 μM MPA separately for 48 h. **F** The apoptosis of Ish-PCMV-YAP/TAZ and Ish-PCMV-Ctrl cells treated with 0, 15, 30 μM MPA separately for 48 h showed by flow cytometry assay. **G** The expression of apoptosis and proliferation protein markers in Ish-PCMV-YAP/TAZ and Ish-PCMV-Ctrl cells treated with 0, 15 μM MPA separately. **H** Statistical graph of (**G**). **I** The migration and invasion capacity of Ish-PCMV-YAP/TAZ and Ish-PCMV-Ctrl cells at 0, 15 μM MPA showed by Transwell assay. **J** The migration capacity of Ish-PCMV-YAP/TAZ and Ish-PCMV-Ctrl cells at 0, 15 μM MPA showed by wound healing assay. *P < 0.05, **P < 0.01, ***P < 0.001, and ****P < 0.0001 and ####*P* < 0.0001.

We established stably Ish-PCMV-YAP/TAZ, Ish-PCMV-YAP, and Ish-PCMV-TAZ cells by transfected lentivirus into Ishikawa cells and investigated transfection efficiency by western blotting and RT-PCR (Fig. 4A–C, Supplementary Fig. 3A, Original Data Fig. 4A, Original Data Supplementary Fig. 3A). Cells that overexpressed both YAP and TAZ were more likely to proliferate and resist apoptosis than cells that overexpressed YAP or TAZ alone, especially in the presence of MPA (Fig. 4D, Supplementary Fig. 3B–D). These results indicated that YAP and TAZ, as a key complex, could better increase the resistance of cells to MPA. We also observed that with elevated concentrations of MPA, DNA synthesis ability was stronger in Ish-PCMV-YAP/TAZ cells than Ish-PCMV-Ctrl cells (Fig. 4E, Supplementary Fig. 3E). By flow cytometry, compared 0 μM and 15 μM or 30 μM MPA treatments, the proportions of apoptosis in Ish-PCMV-Ctrl cells changed more significantly than those in Ish-PCMV-YAP/TAZ cells (Fig. 4F, Supplementary Fig. 3F). The same trend could be observed by detection of proliferation and apoptosis proteins through western blotting assay. We also observed that after MPA treatment the expression of both YAP and TAZ tended to increase in Ish-PCMV-YAP/TAZ and Ish-PCMV-Ctrl cells but the change of YAP and TAZ expression in Ish-PCMV-Ctrl cells was mild (Fig. 4G, H, Original Data Fig. 4G). To discriminate between endogenous and overexpressed YAP and TAZ, we performed western blotting assay to detect flag expression. Results showed that there is little change of overexpressed YAP and TAZ after progestin treatment as the change of flag expression was not obvious. (Supplementary Fig. 3G, Original Data Supplementary Fig. 3G). Besides, Transwell and wound healing assays revealed that overexpression of YAP/TAZ also could increase the ability of invasion and migration in Ishikawa cells and the metastasis ability of Ish-PCMV-Ctrl cells was more significantly inhibited by MPA than that of Ish-PCMV-YAP/TAZ cells (Fig. 4I, J).

Taken above results together, suppression of YAP/TAZ in IshikawaPR cells increased sensitivity to MPA while overexpression of YAP/TAZ in Ishikawa cells increased tolerance to MPA and induced progestin resistance. It is reasonable that YAP/TAZ play a crucial part in proliferation, apoptosis, metastasis and MPA resistance of EC cell lines.

### YAP/TAZ are involved in progestin resistance of EC through PI3K-Akt pathway

**Fig. 5:**
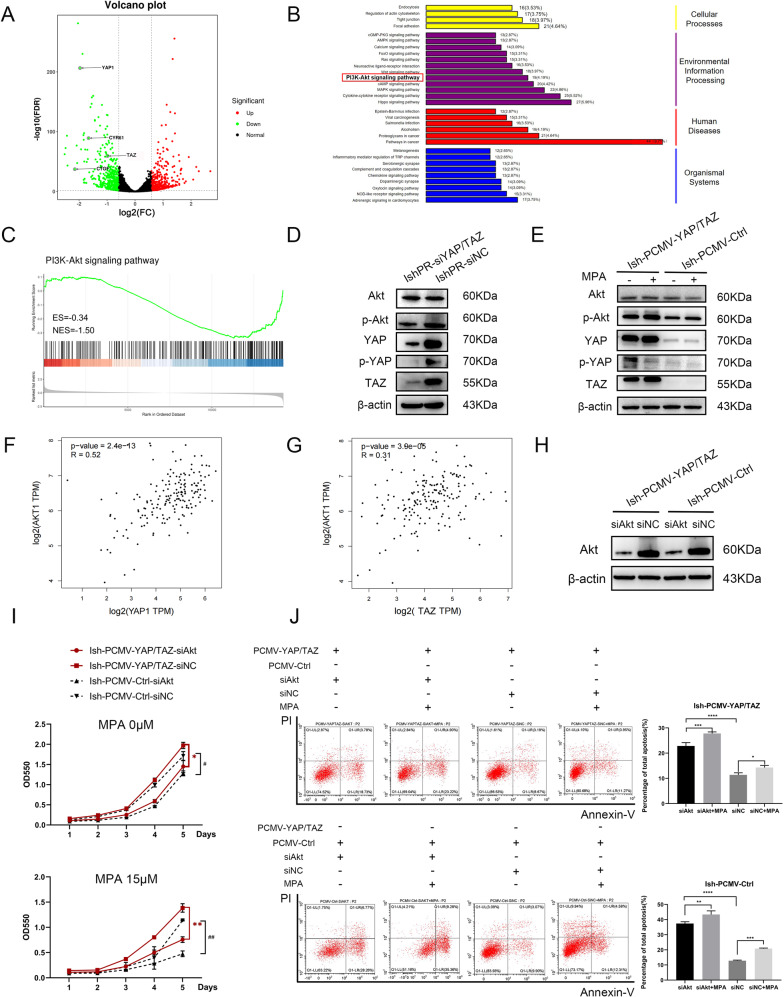
YAP/TAZ are involved in progestin resistance of EC through PI3K-Akt pathway. **A** Volcano plot of RNA-seq from microarray data in IshPR-siYAP/TAZ and IshPR-siNC cells. The green and red dots represent the downregulated and upregulated genes, respectively. **B**, **C** KEGG enrichment analysis and GSEA analysis showing that suppression of YAP/TAZ affected PI3K-Akt pathway. **D** The expression of Akt, p-Akt when knocking down YAP/TAZ in IshikawaPR cells. **E** The expression of Akt, p-Akt with MPA treatment in Ish-PCMV-YAP/TAZ and Ish-PCMV-Ctrl cells. **F**, **G** The correlation of YAP/TAZ and Akt according to GEPIA database. **H** The efficiency of siAkt in Ish-PCMV-YAP/TAZ and Ish-PCMV-Ctrl cells detected by western blotting. **I** The growth curves of Ish-PCMV-YAP/TAZ and Ish-PCMV-Ctrl cells after transfected with siAkt and siNC and treated with 0, 15 μM MPA at 1–5 days showed by MTT assay. *P < 0.05, **P < 0.01 Ish-PCMV-YAP/TAZ-siAkt vs Ish-PCMV-YAP/TAZ-siNC. #*P* < 0.05, ##*P* < 0.01 Ish-PCMV-Ctrl-siAkt vs Ish-PCMV-Ctrl-siNC. **J** The apoptosis of Ish-PCMV-YAP/TAZ and Ish-PCMV-Ctrl cells after transfected with siAkt and siNC and treated with 0, 30 μM MPA for 48 h by flow cytometry assay. *P < 0.05, **P < 0.01, ***P < 0.001, ****P < 0.0001.

To explore the further mechanisms that YAP/TAZ involved in MPA resistance, we conducted next-generation sequencing (NGS) to contrast the differential expression genes between established IshPR-siYAP/TAZ and IshPR-siNC (*n* = 3). Volcano plot showed that there were 463 up-regulated genes and 489 downregulated genes by silencing YAP/TAZ (Fig. 5A). KEGG enrichment analysis and GSEA analysis revealed that knocking down YAP/TAZ inhibited PI3K-Akt pathway which was testified by western blotting (Fig. 5B–D, Original Data Fig. 5D). Furthermore, overexpression of YAP/TAZ also increased the expression level of p-Akt while that of Akt had little change (Fig. 5E, Original Data Fig. 5E). We also found that both YAP and TAZ were positively correlated with Akt in uterine corpus endometrial carcinoma according to GEPIA database (Fig. 5F, G). According to the above results, it is speculated that YAP/TAZ regulated the response of EC cells to MPA through PI3K-Akt pathway. Then we knocked out Akt in Ish-PCMV-YAP/TAZ cells and overexpressed it in IshikawaPR cells to carry out rescue experiments. Firstly, we transfected siAkt and siNC into Ish-PCMV-YAP/TAZ and Ish-PCMV-Ctrl cells respectively for 24 h and the knockout efficiency was detected by western blotting (Fig. 5H, Original Data Fig. 5H). We treated cells with 0 and 15 μM MPA for 1–5 days to visualize cell viability by MTT assay. Survival curves showed that silencing Akt could inhibit the growth of Ish-PCMV-YAP/TAZ cells with or without MPA treatment (Fig. 5I). Next, we found that deficiency of Akt increased the apoptosis proportions of Ish-PCMV-YAP/TAZ cells especially under MPA administration by flow cytometry assay (Fig. 5J). The above results indicated that silencing Akt in Ish-PCMV-YAP/TAZ cells could increase sensitivity to MPA and reverse MPA resistance induced by overexpression of YAP/TAZ. We also performed rescue experiments in IshikawaPR cells treated with verteporfin and overexpressing Akt. Results showed that IshPR-PCMV-Akt cells grew faster than control cells especially with the treatment of Verteporfin and MPA. Flow cytometry assay indicated that with both Verteporfin and MPA administration, overexpression of Akt could rescue the apoptosis proportions of IshikawaPR cells (Supplementary Fig. 4B–D, Original Data Supplementary Fig. 4B). Therefore, we made a conclusion that YAP/TAZ promoted progestin resistance through regulating PI3K-Akt pathway in EC.

### Verteporfin enhances sensitivity of IshikawaPR cells to progestin by reducing the expression of YAP/TAZ in vivo and vitro

**Fig. 6:**
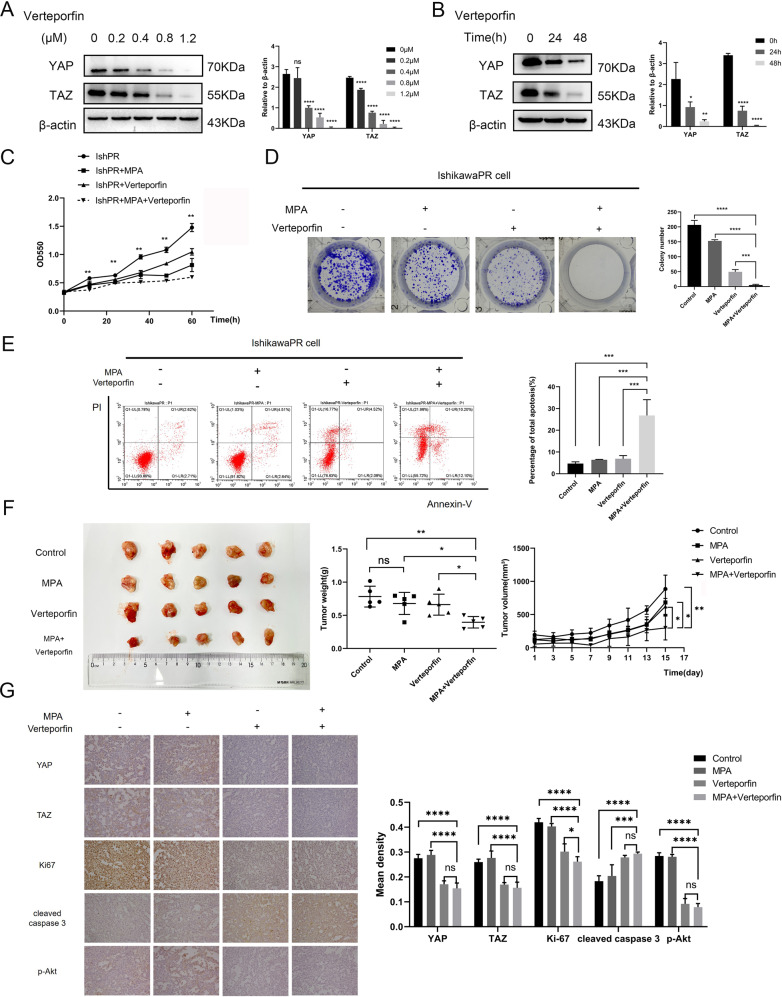
Combination of Verteporfin and MPA increases the lethal effects on EC in vivo and vitro. **A**, **B** The inhibition of YAP/TAZ by Verteporfin in dose-dependent and time-dependent manners by western blotting assay. **C** The growth curves of IshikawaPR cells after treated with medium, MPA, Verteporfin, or MPA plus Verteporfin. **P < 0.01 IshikawaPR cells treated with medium vs IshikawaPR cells treated with MPA plus Verteporfin. **D** The colony formation ability of IshikawaPR cells with treatment of medium, MPA, Verteporfin, and MPA plus Verteporfin. **E** The apoptosis of IshikawaPR cells with treatment of medium, MPA, Verteporfin or MPA plus Verteporfin detected by flow cytometry assay. **F** The effects of Verteporfin, MPA, and MPA plus Verteporfin on tumors growth in vivo. **G** Representative images of IHC staining of YAP, TAZ, Ki67, cleaved caspase 3, and p-Akt in tumor tissues. *P < 0.05, **P < 0.01, ***P < 0.001 and ****P < 0.0001.

Consistent with previous findings, western blotting revealed Verteporfin inhibited the expression of YAP/TAZ in dose-dependent and time-dependent manner in IshikawaPR cells (Fig. 6A, B, Original Data Fig. 6A, B). Combination of Verteporfin and MPA could inhibit the proliferation of IshikawaPR cells more obviously than Verteporfin or MPA alone (Fig. 6C). There was the same tendency showed by colony formation assay in the long-term effects (Fig. 6D). We calculated the combination index (CI) of combination MPA and Verteporfin as shown in Supplementary Table 1. The value indicated a slight to moderate synergism between MPA and Verteporfin. What’s more, flow cytometry revealed combination of Verteporfin and MPA increased cell apoptosis significantly (Fig. 6E). Subsequently, we established xenograft models in nude mice using IshikawaPR cells. Results showed that with the prolongation of administration time, the volumes of tumors in Verteporfin plus MPA group were the smallest in four groups, indicating that compared with Verteporfin or MPA alone, combined treatment significantly prevented tumor from growing (Fig. 6F). Meanwhile, YAP, TAZ, Ki67, cleaved caspase 3, and p-Akt of tumors were stained by IHC. Results showed that the expression levels of YAP, TAZ, Ki67 were lowest and cleaved caspase 3 was highest in the combined group comparing with other groups. The expression levels of p-Akt in the four groups indicated that the inhibition of YAP/TAZ by Verteporfin could suppress the activation of Akt (Fig. 6G). To sum up, Verteporfin can mimic the effects of siYAP/TAZ and combination of Verteporfin and MPA increases the lethal effect on EC in vivo and vitro.

## Discussion

Increasing morbidity and mortality of endometrial carcinoma have been troubling patients and doctors around the world. For young women with EC, it is urgent to find a conservative treatment that preserves fertility. According to latest NCCN guidelines(version1.2022), there are a limited group of women able to choose fertility-sparing therapy which is based on continuous progestin administration. Moreover, a part of the patients will fail to response to hormonal therapy or become recurrent because of progestin resistance [1, 3, 5]. Recent years many researchers have tried to find out mechanisms involved in progestin resistance. According to previous studies, PI3K-Akt pathway, fas/fasl pathway and EMT pathway as well as some molecules like DACH1, SREBP1, and miR-92a are certified to induce resistant process [8, 32–34].

In order to find out the underlying mechanisms of MPA resistance, we performed microarray analysis as well as DEGs and KEGG enrichment analysis between Ishikawa cells and IshikawaPR cells. It showed that Hippo pathway significantly changed in IshikawaPR cells. YAP/TAZ are widely regarded as core downstream transcription coactivators of Hippo pathway. When Hippo pathway are off, YAP/TAZ will dephosphorylate shuttling from cytoplasm to nucleus. In the nucleus, they can bind the transcription factors and promote the expression of their target genes so as to regulate proliferation, survival, inflammation, metabolism and oncogene activity [19, 24, 35, 36]. It has been reported that YAP/TAZ play a key role in progression of various cancers and YAP or TAZ solely may not be sufficient enough to initiate cancers [37]. In EC, previous studies showed that silencing YAP/TAZ inhibited cell growth, migration and invasion and induced cell death more dramatically than siYAP or siTAZ separately [28]. Additionally, a growing number of research indicates that activation of YAP/TAZ mediates resistance to cancer therapy [19]. Our research found that the expression levels of YAP/TAZ were upregulated in the progestin resistant cells and tissues. Progestin can inhibit cancer cell proliferation through binding to PGR and a reduction in the number or decreased availability of PGR leads to progestin resistance [38]. So, we analyzed the correlation between YAP/TAZ and PGR in progestin resistant tissues and found that there were negative correlations between nuclear YAP/TAZ and PGR. Our results also revealed that PGR decreased in IshikawaPR cells. Based on the results, we made a preliminary conclusion that YAP/TAZ may play a key role in progestin resistance of EC.

There are many studies indicating that YAP and TAZ as a whole are involved in the drug resistance process and targeting YAP and TAZ could overcome drug resistance of tumors [10, 24, 25]. In order to verify how YAP/TAZ function in progestin resistance of EC, we performed a series of experiments in vitro. Our preliminary studies proved that silencing YAP/TAZ together could increase sensitivity of IshikawaPR cells to progestin more significantly compared with silencing YAP or TAZ alone while overexpression of both YAP and TAZ could better increase the resistance of cells to MPA than overexpression of YAP and TAZ respectively. So, we speculated that YAP and TAZ as a key complex could play a vital role in progestin resistance of EC. Next, our results revealed that with elevated MPA concentration treatment, cells knocking down YAP and TAZ were more sensitive to MPA and cells overexpressing YAP and TAZ were more resistant to MPA than control cells, which is consistent with our previous speculation. Similar results can be found in hepatocellular carcinoma and tamoxifen-resistant MCF7 breast cancer [25, 39]. Taken together, YAP/TAZ, as a promoter of tumors, is essential in the process of drug resistance.

PI3K-Akt pathway is activated in many tumors and takes part in cell survival, metastasis and metabolism. Studies [10, 40] have shown that PI3K-Akt plays a key link in multidrug resistance, but in some cases activation of PI3K-Akt pathway alone is not sufficient to lead to the development of tumor chemoresistance. PI3K-Akt signaling pathway is influenced by a large number of different inputs that are involved in chemotherapy resistance. It was reported that the PI3K-Akt signaling pathway promotes progestin resistance in endometrial cancer [8, 34], which is consistent with our KEGG enrichment analysis (showed in Fig. 1A) and our experimental results (showed in Supplementary Fig. 4A, Original Data Supplementary Fig. 4A). The KEGG enrichment analysis also reported that in IshikawaPR cells PI3K-Akt pathway was much more affected than other pathways. A previous study showed that Nrf2 plays an important role in the development of progestin resistance while Nrf2 could lead to the progression of cell cycle by activating Akt pathway [41]. It was also reported that chlorpromazine reverses progestin resistance by inhibiting PI3K-Akt pathway [42]. So PI3K-Akt pathway may be a key link in regulating progestin resistance of EC. Research had also suggested that YAP/TAZ participate in cellular activity by regulating PI3K-Akt pathway through different ways [28]. Based on the next-generation sequencing (NGS) results and these studies, we speculated that YAP/TAZ promote progestin resistance by regulating PI3K-Akt pathway in EC. Consistent with our analysis, we found that deletion or overexpression of YAP/TAZ would result in corresponding changes of p-Akt. The further rescue experiments indicated that suppression of Akt in Ishikawa cell and overexpression of Akt in IshikawaPR cells could rescue the corresponding phenotypes. Based on the above results, we concluded that YAP/TAZ induce progestin resistance of EC by regulating PI3K-Akt pathway. It is also reasonable to assume that in the process of progestin resistance PI3K-Akt pathway may be affected by Hippo pathway as well as a number of other synergistic pathways, which need to be further explored.

Finally, we used YAP/TAZ specific inhibitor Verteporfin to further confirm the role of YAP/TAZ involving in progestin resistance of EC. Verteporfin has been reported to inhibit cell proliferation by targeting YAP/TAZ in tumors like EC and pancreatic ductal adenocarcinoma [28, 43]. However, there are few studies about the effects of Verteporfin on progestin resistance of EC. Our study confirmed that Verteporfin, mimicking knocking down YAP/TAZ, decreased cell viability and reversed resistance to progestin in EC by in vivo and vitro experiments. And we also found combination of Verteporfin and MPA could suppress the growth of tumors more obviously, which implied that Verteporfin could be used clinically to improve progestin resistance of EC patients.

In summary, YAP/TAZ can promote progestin resistance through PI3K-Akt pathway in endometrial carcinoma. Verteporfin, an inhibitor of YAP/TAZ, is also confirmed to mimic the action of knocking YAP and TAZ down and is able to enhance the sensitivity of endometrial carcinoma cells to progestin treatment. Progestin in combination with Verteporfin may be an alternative conservative treatment for endometrial carcinoma.

## Materials and methods

### Patients and tissue samples

The patients who had ever undergone conservative treatment with progestin were collected from Qilu hospital, Shandong University. The tissue samples were obtained from the Pathology Department of Qilu hospital, which were diagnosed according to the latest National Comprehensive Cancer Network (NCCN) guidelines. The patients had regularly received medroxyprogesterone acetate (MPA) for at least 2 cycles, more than 6 months, who were divided into complete response group (CR, *n* = 3); partial response group (PR, *n* = 9); progressive disease/stable disease group (PD/SD, *n* = 29). CR group means the residual lesions of hyperplasia or cancer were less than 5% of the tissues; PR means less than 50% while PD/SD means the lesions were not diminished or even larger than those before treatment.

### Antibodies and agents

Antibodies for YAP1(ab76252), PGR(ab23085), and TBP (ab818) were purchased from Abcam (Cambridge, UK); TAZ (#83669), β-actin(#4970), MST1(#3682), parp(#2772), cleaved caspase 3(#9664), cleaved caspase 7(#8438), cleaved caspase 9(#20750), bax(#2772), and CyclinD1(#2978), Akt(#4691), p-Akt(#13038), anti-flag(#14793), anti-rabbit IgG(#7074), anti-mouse IgG(#7076) were from Cell Signaling Technology (Danvers, MA,USA). Antibody for LATS1(#sc-398560) was from Santa Cruz Biotechnology (Santa Cruz, CA, USA). Antibodies for p-MST1(AP0906), p-LATS1(AP0904), p-YAP(AP0489) were from ABcolonal (Wuhan, China). Medroxyprogesterone acetate, abbreviated to MPA (#ab142633) was purchased from Abcam and Verteporfin (CL318952) was purchased from MedChem Express (Monmouth Junction, NJ, USA).

### Cell lines and cell culture

We purchased Ishikawa cells from Shanghai Zhong Qiao Xin Zhou Biotechnology Co., Ltd. MPA resistant cells, also named IshikawaPR cells, were obtained by exposed in MPA with increasing concentrations [44]. Ishikawa cells were cultured in Dulbecco’s modified Eagle’s medium (DMEM) and IshikawaPR cells were cultured in RPMI 1640 medium at 37 °C in a 5% CO_2_ humidified incubator. Both medium contained 10% fetal bovine serum (FBS) and 1% 100 U/ml penicillin.

### MTT assay

MTT, 3-(4, 5-dimethylthiazol-2-yl)-2, 5-diphenyl-tetrazolium bromide, was applied to analyze cell viability and 50% inhibitory concentrations (IC50). Totally, 0.2 × 10^4^ cells per well were seeded into 96-well plate and overnight the cells were incubated with different concentrations of MPA (0, 15, 30, 45, 60, 90, and 120 μM) for 48 h. Then 10 μL MTT (5 mg/mL in PBS) was added into each well at 37 °C and 4–6 h later, formazan crystals were dissolved in 100 μL dimethylsulfoxide (Sigma‐Aldrich, St Louis, MO, USA). The absorbance was measured at 550 nm wavelength.

### EDU incorporation assay

EDU, 5-ethynyl-20-deoxyuridine, incorporation assay kit (Ribobio, Guangzhou, China) was used to detect the proliferation of cells. Totally, 1 × 10^4^ cells were seeded into each well of 96-well plate and incubated overnight. Then the medium was replaced by fresh one containing 0, 45, 90 μM or 0, 15, 30 μM MPA for 48 h. According to manufacturer’s instructions, 50 μM EDU was added to fix, permeate, stain the cells and finally 1× Hoechst was used to stain the cell nucleus for 30 min before detecting by fluorescence microscopy. The data were obtained from at least five captured views.

### Western blotting

Cells was lysed by RIPA lysis buffer (Beyotin, Beijing, China), 1%PMSF and 1% NaF for 30 min at 4 °C. After separating by 10% SDS-PAGE, transferring to PVDF-membranes (Merck Millipore, Burlington, MA, USA) and blocking by blocking buffer, the membranes were put into specific primary antibodies for over 12 h at 4 °C. Then, membranes were incubated for 1.5 h in secondary antibodies at room temperature and finally protein bands were visualized by Image Quant LAS4000 (General Electric Company, Boston, MA, USA) and quantified by ImageJ software. β-actin and TBP were used as endogenous controls.

### Flow cytometry assay

After treated with 0, 45, 90 μM or 0, 15, 30 μM MPA for 48 h, cells were digested with trypsin without EDTA and suspended by 100 μL 1× binding buffer. 5 μL Annexin-V/APC and 5 μL 7-AAD (Annexin V APC apoptosis kit from BioGems, 62700-80, USA) were added into each tube. Fifteen minutes later cells were detected and the apoptosis data was analyzed by CytoFLEX flow cytometry (Beckman Coulter, USA) and CytExpert Software.

### RT-PCR

RNA of cells was extracted using Trizol reagent (Invitrogen, Carlsbad, CA, USA). Concentration and purity were measured by spectrophotometer (Thermo Fisher Scientific Inc., MA, USA). Totally, 500 ng RNA was reverse transcribed into cDNA by M-MLV system (Cat no. C28025-011; Invitrogen, China). PCR reactions were performed by the 7900HT Fast Real-Time PCR System (Applied Biosystems, Waltham, MA, USA) with SYBR-green (TAKARA, Japan) in a 10 μl reaction mixture. The primers are shown in additional information (Supplementary Table 2).

### Wound healing assay

Totally, 5 × 10^4^ cells were seeded in 12-well plate and incubated overnight. Medium was replaced by fresh one with 0, 45, 90 μM or 0, 15 or 30 μM MPA and 48 h later 100-μL pipette tips were used to scratch wounds. At 0 h, 24 h, 48 h, 72 h wounds were photographed and open wound areas were measured by ImageJ software.

### Transwell assay

After dealt with different concentrations of MPA for 48 h, 12 × 10^4^ cells were suspended by serum-free medium and added into the upper Transwell chamber (Corning Life Sciences, Corning, NY, USA). The lower chambers were filled with 700 μl medium with 15% FBS. Cells penetrating through the membrane were fixed by methanol for 30 min after incubated 18 h at 37 °C and stained by 1% crystal violet (Beyotime, Beijing, China) for 30 min. Invasion is similar to migration, but the cells’ number seeded was up to 15 × 10^4^ and 60 μL mixture of Matrigel (BD Biosciences, USA) and medium was put onto the membrane. The penetrating time for invasion was prolonged to 32 h. Images were captured by fluorescence microscopy.

### Colony formation assay

One thousands cells were seeded into six-well plate. After incubated for 7 days, cells were treated by different concentrations drugs for another 7 days. Colony formations were collected by fixing cells with methanol for 30 min and staining them with crystal violet for 30 min.

### RNA interference (RNAi) and Lentivirus packaging and infection

Specific siRNA and negative control siRNA were purchased from GenePharma (Shanghai, China). At a 20–30% of cell density, siRNAs were transfected into cells with Lipofectamine 3000 reagent (11668-019; Invitrogen, China) for 24 h and the silencing efficiency was detected by western blotting and RT-PCR. The information of targeting sequences of RNAi is shown in Supplementary Table 3.

The lentivirus of YAP/TAZ overexpression were obtained from GenePharma. Full-length DNA of human YAP (NM_001130145) and TAZ (NM_015472) was cloned into the vector GV358 and GV492, respectively. The vector constructs are shown in Supplementary Table 4. Ishikawa cell lines were transfected simultaneously with lentivirus of YAP and TAZ according to manufacturer’s protocols. The infected stable cells were selected by 5 μg/ml puromycin (Solarbio, Beijing, China) for 5 days.

### Immunohistochemistry (IHC)

Paraffin sections were baked for 2 h at 65 °C and dewaxed in xylene and dehydrated in a series of graded ethanol solutions starting with 100% to 50% ethanol. Then antigen retrievals were performed by immersing the slides in IHC citrate buffer. Endogenous peroxidase activity and non-specific antigens were blocked with H_2_O_2_ and serum respectively. The tissues were incubated with specific antibodies to YAP1(1:400), TAZ (1:50), PGR (1:50), cleaved caspase 3 (1:400), Ki-67(1:500), p-Akt (1:500) overnight and then exposed with secondary antibody. After that visualization was performed with chromagen 3,3’-diaminobenzidine (DAB) for 2–10 min. Finally, it is counterstained with hematoxylin, dehydrated by alcohol, and cleaned with xylene before installation. Image-Pro Plus 6.0 was used to quantify the mean intensities of IHC staining.

The intensity of nuclear YAP and TAZ was graded as follows: week: 1; moderate: 2; strong: 3. The score = (percentage of weak intensity × 1) + (percentage of moderate intensity × 2) + (percentage of strong intensity × 3).

### Xenograft model in nude mice

Tumor xenograft experiments had been approved by Laboratory Animal Ethical and Welfare Committee of Shandong University Cheeloo College of Medicine. The BALB/c nude mice (female, aged 4 weeks, 14.3 ± 1.48 g), were purchased from Beijing Vital River Laboratory Animal Technology Co., Ltd., and fed in SPF breeding units. Totally, 1 × 10^7^ cells were suspended in 100 μL PBS and subcutaneously injected into each mouse through the left armpits. When the diameters of xenograft tumors reached approximately 5 mm, mice were divided in a randomized block design into four groups to receive respectively normal saline, MPA (100 mg/kg/day), Verteporfin(40 mg/kg/day) or MPA plus Verteporfin every other day. The weight of mice and volume of tumors were measured every 2 days. Volume calculation formula is: tumor volume = width^2^ × length/2.

### Calculation of combination index

Combination index (CI) value can be used to determine the degree of drug interaction. CI < 1 means synergistic effect; CI = 1 means additive effect; CI > 1 means antagonistic effect. CalcuSyn Version 2.0 was used to analyze CI value according to Chou–Talalay theory.

### Statistical analysis

All experiments were performed independently at least three times and data in the studies was analyzed for normality and lognormality by Graphpad Prism 8.0 Version and expressed as mean ± SD. Statistical significance was defined as *P* < 0.05 using Student’s *t* tests, one‐way ANOVA, two‐way ANOVA and Mann–Whitney test.

## Supplementary information

Author Contribution Statement

Supplementary Figure legends

Supplementary Figure 1

Supplementary Figure 2

Supplementary Figure 3

Supplementary Figure 4

Supplementary Tables

Supplementary information

Original Data File

Original Data File

## Data Availability

The datasets used and analyzed during the current study are available from the corresponding author on reasonable request.

## References

[1] Lu KH, Broaddus RR (2020). Endometrial cancer. N. Engl J Med..

[2] Gracia CR, Jeruss JS (2013). Lives in the balance: women with cancer and the right to fertility care. J Clin Oncol..

[3] Gunderson CC, Fader AN, Carson KA, Bristow RE (2012). Oncologic and reproductive outcomes with progestin therapy in women with endometrial hyperplasia and grade 1 adenocarcinoma: a systematic review. Gynecol Oncol..

[4] Hubbs JL, Saig RM, Abaid LN, Bae-Jump VL, Gehrig PA (2013). Systemic and local hormone therapy for endometrial hyperplasia and early adenocarcinoma. Obstet Gynecol..

[5] Ushijima K, Yahata H, Yoshikawa H, Konishi I, Yasugi T, Saito T (2007). Multicenter phase II study of fertility-sparing treatment with medroxyprogesterone acetate for endometrial carcinoma and atypical hyperplasia in young women. J Clin Oncol..

[6] Park JY, Kim DY, Kim JH, Kim YM, Kim KR, Kim YT (2013). Long-term oncologic outcomes after fertility-sparing management using oral progestin for young women with endometrial cancer (KGOG 2002). Eur J Cancer.

[7] Hahn HS, Yoon SG, Hong JS, Hong SR, Park SJ, Lim JY (2009). Conservative treatment with progestin and pregnancy outcomes in endometrial cancer. Int J Gynecol Cancer.

[8] Gu C, Zhang Z, Yu Y, Liu Y, Zhao F, Yin L (2011). Inhibiting the PI3K/Akt pathway reversed progestin resistance in endometrial cancer. Cancer Sci..

[9] Huang J, Wu S, Barrera J, Matthews K, Pan D (2005). The Hippo signaling pathway coordinately regulates cell proliferation and apoptosis by inactivating Yorkie, the Drosophila Homolog of YAP. Cell.

[10] Lo Sardo F, Canu V, Maugeri-Sacca M, Strano S, Blandino G (2022). YAP and TAZ: monocorial and bicorial transcriptional co-activators in human cancers. Biochim Biophys Acta Rev Cancer.

[11] Vlug EJ, van de Ven RA, Vermeulen JF, Bult P, van Diest PJ, Derksen PW (2013). Nuclear localization of the transcriptional coactivator YAP is associated with invasive lobular breast cancer. Cell Oncol..

[12] Skibinski A, Breindel JL, Prat A, Galvan P, Smith E, Rolfs A (2014). The Hippo transducer TAZ interacts with the SWI/SNF complex to regulate breast epithelial lineage commitment. Cell Rep..

[13] Guo Y, Pan Q, Zhang J, Xu X, Liu X, Wang Q (2015). Functional and clinical evidence that TAZ is a candidate oncogene in hepatocellular carcinoma. J Cell Biochem..

[14] Chen S, Wang H, Huang YF, Li ML, Cheng JH, Hu P (2017). WW domain-binding protein 2: an adaptor protein closely linked to the development of breast cancer. Mol Cancer.

[15] Han SX, Bai E, Jin GH, He CC, Guo XJ, Wang LJ (2014). Expression and clinical significance of YAP, TAZ, and AREG in hepatocellular carcinoma. J Immunol Res..

[16] Lau AN, Curtis SJ, Fillmore CM, Rowbotham SP, Mohseni M, Wagner DE (2014). Tumor-propagating cells and Yap/Taz activity contribute to lung tumor progression and metastasis. EMBO J..

[17] Noguchi S, Saito A, Horie M, Mikami Y, Suzuki HI, Morishita Y (2014). An integrative analysis of the tumorigenic role of TAZ in human non-small cell lung cancer. Clin Cancer Res..

[18] Wang Y, Dong Q, Zhang Q, Li Z, Wang E, Qiu X (2010). Overexpression of yes-associated protein contributes to progression and poor prognosis of non-small-cell lung cancer. Cancer Sci..

[19] Zanconato F, Cordenonsi M, Piccolo S (2016). YAP/TAZ at the roots of cancer. Cancer Cell.

[20] Edgar BA (2006). From cell structure to transcription: Hippo forges a new path. Cell.

[21] Tang Y, Feinberg T, Keller ET, Li XY, Weiss SJ (2016). Snail/Slug binding interactions with YAP/TAZ control skeletal stem cell self-renewal and differentiation. Nat Cell Biol.

[22] Zanconato F, Forcato M, Battilana G, Azzolin L, Quaranta E, Bodega B (2015). Genome-wide association between YAP/TAZ/TEAD and AP-1 at enhancers drives oncogenic growth. Nat Cell Biol.

[23] Dong J, Feldmann G, Huang J, Wu S, Zhang N, Comerford SA (2007). Elucidation of a universal size-control mechanism in Drosophila and mammals. Cell.

[24] Nguyen CDK, Yi C (2019). YAP/TAZ signaling and resistance to cancer therapy. Trends Cancer.

[25] Kim YJ, Jang SK, Hong SE, Park CS, Seong MK, Kim HA (2022). Knockdown of YAP/TAZ sensitizes tamoxifen-resistant MCF7 breast cancer cells. Biochem Biophys Res Commun..

[26] Romero-Perez L, Garcia-Sanz P, Mota A, Leskela S, Hergueta-Redondo M, Diaz-Martin J (2015). A role for the transducer of the Hippo pathway, TAZ, in the development of aggressive types of endometrial cancer. Mod Pathol..

[27] Tsujiura M, Mazack V, Sudol M, Kaspar HG, Nash J, Carey DJ (2014). Yes-associated protein (YAP) modulates oncogenic features and radiation sensitivity in endometrial cancer. PLoS ONE.

[28] Wang C, Gu C, Jeong KJ, Zhang D, Guo W, Lu Y (2017). YAP/TAZ-mediated upregulation of GAB2 leads to increased sensitivity to growth factor-induced activation of the PI3K pathway. Cancer Res..

[29] Larsen M, Schmidt-Erfurth U, Lanzetta P, Wolf S, Simader C, Tokaji E (2012). Verteporfin plus ranibizumab for choroidal neovascularization in age-related macular degeneration: twelve-month MONT BLANC study results. Ophthalmology.

[30] Hwang SM, Jin M, Shin YH, Ki Choi S, Namkoong E, Kim M (2014). Role of LPA and the Hippo pathway on apoptosis in salivary gland epithelial cells. Exp Mol Med..

[31] Zhao S, Li G, Yang L, Li L, Li H (2013). Response-specific progestin resistance in a newly characterized Ishikawa human endometrial cancer subcell line resulting from long-term exposure to medroxyprogesterone acetate. Oncol Lett..

[32] Zhou Q, Li W, Kong D, Liu Z, Shi Z, Ma X (2019). DACH1 suppresses epithelial to mesenchymal transition (EMT) through Notch1 pathway and reverses progestin resistance in endometrial carcinoma. Cancer Med..

[33] Ma X, Zhao T, Yan H, Guo K, Liu Z, Wei L (2021). Fatostatin reverses progesterone resistance by inhibiting the SREBP1-NF-kappaB pathway in endometrial carcinoma. Cell Death Dis..

[34] Liu H, Zhang L, Zhang X, Cui Z (2017). PI3K/AKT/mTOR pathway promotes progestin resistance in endometrial cancer cells by inhibition of autophagy. Onco Targets Ther.

[35] Piccolo S, Dupont S, Cordenonsi M (2014). The biology of YAP/TAZ: hippo signaling and beyond. Physiol Rev..

[36] Ouyang T, Meng W, Li M, Hong T, Zhang N (2020). Recent advances of the Hippo/YAP signaling pathway in brain development and glioma. Cell Mol Neurobiol..

[37] Dobrokhotov O, Samsonov M, Sokabe M, Hirata H (2018). Mechanoregulation and pathology of YAP/TAZ via Hippo and non-Hippo mechanisms. Clin Transl Med..

[38] Wang S, Pudney J, Song J, Mor G, Schwartz PE, Zheng W (2003). Mechanisms involved in the evolution of progestin resistance in human endometrial hyperplasia—precursor of endometrial cancer. Gynecologic Oncol..

[39] Gao R, Kalathur RKR, Coto-Llerena M, Ercan C, Buechel D, Shuang S (2021). YAP/TAZ and ATF4 drive resistance to Sorafenib in hepatocellular carcinoma by preventing ferroptosis. EMBO Mol Med..

[40] Liu R, Chen Y, Liu G, Li C, Song Y, Cao Z (2020). PI3K/AKT pathway as a key link modulates the multidrug resistance of cancers. Cell Death Dis..

[41] Zimta AA, Cenariu D, Irimie A, Magdo L, Nabavi SM, Atanasov AG (2019). The role of Nrf2 activity in cancer development and progression. Cancers (Basel).

[42] Cui Y, Wu H, Yang L, Huang T, Li J, Gong X (2021). Chlorpromazine sensitizes progestin-resistant endometrial cancer cells to MPA by upregulating PRB. Front Oncol..

[43] Wei H, Wang F, Wang Y, Li T, Xiu P, Zhong J (2017). Verteporfin suppresses cell survival, angiogenesis and vasculogenic mimicry of pancreatic ductal adenocarcinoma via disrupting the YAP-TEAD complex. Cancer Sci..

[44] Wang Y, Zhang L, Che X, Li W, Liu Z, Jiang J (2018). Roles of SIRT1/FoxO1/SREBP-1 in the development of progestin resistance in endometrial cancer. Arch Gynecol Obstet..

