# Supplementary material for: Verteporfin reverses progestin resistance through YAP/TAZ-PI3K-Akt pathway in endometrial carcinoma
Source: Cell Death Discov. 2023 Jan 25;9:30. doi: 10.1038/s41420-023-01319-y (PMC9873621; doi:10.1038/s41420-023-01319-y)
Supplement: Supplementary file 1 — Author Contribution Statement [file 41420_2023_1319_MOESM1_ESM.docx]

**Contribution to preparation of manuscript:**

Jie Jiang, Lina Wei and Xiaohong Ma designed the study. Lina Wei, Xiaohong Ma, Yixin Hou participated in performing and analyzing the experiments. Chunping Qiu, Tianyi Zhao and Zhiming Liu analyzed the data and reviewed the paper. Rui Sun, Yao Liu, Ziyi Qiu participated in conducting the experiments. All authors read the paper and approved the final manuscript.

**Detailed preparation of figures:**

In Figure 1-6, Lina Wei and Xiaohong Ma generated the clinical data and analyzed the data; Yixin Hou and Tianyi Zhao generated the immunohistochemistry data and labelled the image; Lina Wei and Ziyi Qiu generated the EDU and Western blotting data; Lina Wei, Xiaohong Ma and Yao Liu analyzed the bioinformatics data; Lina Wei and Rui Sun conducted the animal experiments; Lina Wei, Zhiming Liu and Chunping Qiu generated the remaining data and assembled the figures.
