# Supplementary material for: Verteporfin reverses progestin resistance through YAP/TAZ-PI3K-Akt pathway in endometrial carcinoma
Source: Cell Death Discov. 2023 Jan 25;9:30. doi: 10.1038/s41420-023-01319-y (PMC9873621; doi:10.1038/s41420-023-01319-y)
Supplement: Supplementary file 2 — Supplementary Figure legends [file 41420_2023_1319_MOESM2_ESM.docx]

**Supplementary Figure 1**

1A. The expression of cytosol/nucleus fractions of IshikawaPR cells and Ishikawa cells by western blotting assay.

1B. The transcription levels of the target genes, CTGF, CYR61 and ANKRD1, in IshikawaPR cells and Ishikawa cells by RT-PCR.

1C. Nuclear expression of YAP and cytoplasmic expression of p-YAP in IshikawaPR cell with different concentrations of MPA by western blotting.

1D. Nuclear expression of YAP and cytoplasmic expression of p-YAP in IshikawaPR cell at different time points with fixed concentration of MPA by western blotting.

**Supplementary Figure 2**

2A. The efficiency of silencing YAP/TAZ, YAP and TAZ respectively in IshikawaPR cells detected by western blotting assay.

2B. The growth curves of IshPR-siYAP/TAZ, IshPR-siYAP, IshPR-siTAZ and IshPR-siNC cells at 0, 30μM MPA examined by MTT assay at 1-5 days. *P < 0.05, **P < 0.01, ***P < 0.001, ****P < 0.0001 IshPR-siYAP/TAZ cells vs IshPR-siNC.

2C. EDU incorporation assay of IshPR-siYAP/TAZ, IshPR-siYAP, IshPR-siTAZ and IshPR-siNC after treatment with 0, 45μM MPA separately for 48h.

2D. The apoptosis of IshPR-siYAP/TAZ, IshPR-siYAP, IshPR-siTAZ and IshPR-siNC after treatment with 0, 45μM MPA separately for 48h showed by flow cytometry assay.

2E. Statistical graph of EDU incorporation assay in Figure 3E.

2F. Statistical graph of flow cytometry assay in Figure 3F.

**Supplementary Figure 3**

3A. The efficiency of overexpressing YAP/TAZ, YAP and TAZ respectively in Ishikawa cells detected by western blotting assay.

3B. The growth curves of Ish-PCMV-YAP/TAZ, Ish-PCMV-YAP, Ish-PCMV-TAZ and Ish-PCMV-Ctrl cells at 0, 15μM MPA examined by MTT assay at 1-5 days. *P < 0.05, **P < 0.01, ***P < 0.001, ****P < 0.0001 Ish-PCMV-YAP/TAZ cells vs Ish-PCMV-Ctrl cells.

3C. EDU incorporation assay of Ish-PCMV-YAP/TAZ, Ish-PCMV-YAP, Ish-PCMV-TAZ and Ish-PCMV-Ctrl cells after treatment with 0, 30μM MPA separately for 48h.

3D. The apoptosis of Ish-PCMV-YAP/TAZ, Ish-PCMV-YAP, Ish-PCMV-TAZ and Ish-PCMV-Ctrl cells after treatment with 0, 30μM MPA separately for 48h showed by flow cytometry assay.

3E. Statistical graph of EDU incorporation assay in Figure 4E.

3F. Statistical graph of flow cytometry assay in Figure 4F.

3G. The expression of flag in Ish-PCMV-YAP/TAZ and Ish-PCMV-Ctrl cells after 0, 30μM MPA treatment for 48h by western blotting assay.

**Supplementary Figure 4**

4A. The expression of Akt and p-Akt in IshikawaPR cells after treatment with 0, 45μM MPA by western blotting assay.

4B. The efficiency of overexpressing Akt in IshikawaPR cells detected by western blotting assay.

4C. The growth curves of IshPR-PCMV-Akt and IshPR-PCMV-Akt-Ctrl cells with MPA or/and Verteporfin at 1-5 days showed by MTT assay. *P < 0.05, **P < 0.01, ***P < 0.001 IshPR-PCMV-Akt vs IshPR-PCMV-Akt-Ctrl. #P < 0.05, ##P < 0.01, ###P < 0.001 IshPR-Verteporfin-PCMV-Akt vs IshPR-Verteporfin-PCMV-Akt-Ctrl.

4D. The apoptosis of IshPR-PCMV-Akt and IshPR-PCMV-Akt-Ctrl cells with MPA or/and Verteporfin by flow cytometry assay.

*P < 0.05, **P < 0.01, ***P < 0.001, ****P < 0.0001.
