# Supplementary figures and images for: Verteporfin reverses progestin resistance through YAP/TAZ-PI3K-Akt pathway in endometrial carcinoma
Source: Cell Death Discov. 2023 Jan 25;9:30. doi: 10.1038/s41420-023-01319-y (PMC9873621; doi:10.1038/s41420-023-01319-y)

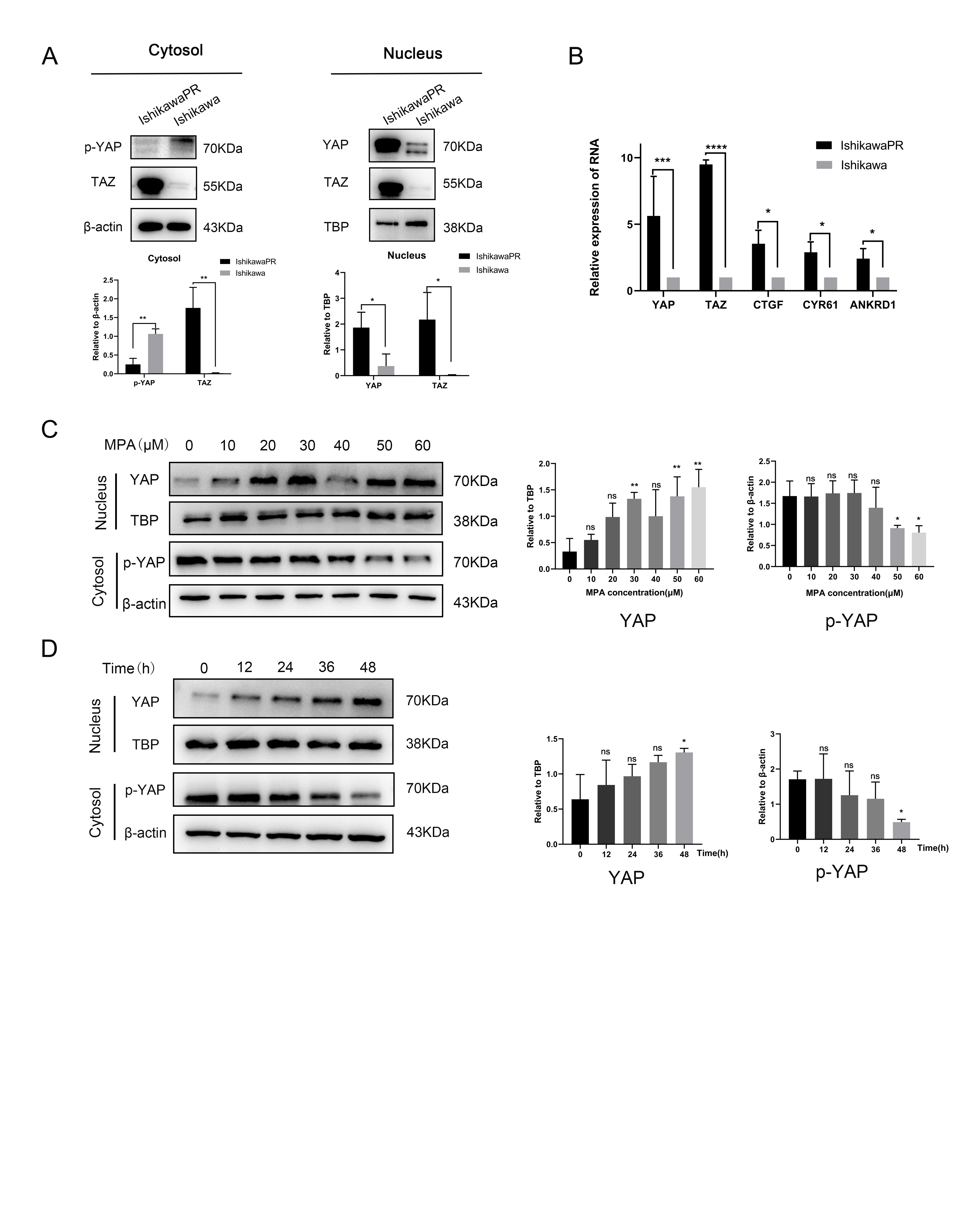

Supplement: Supplementary file 3 — Supplementary Figure 1 [file 41420_2023_1319_MOESM3_ESM.jpg]

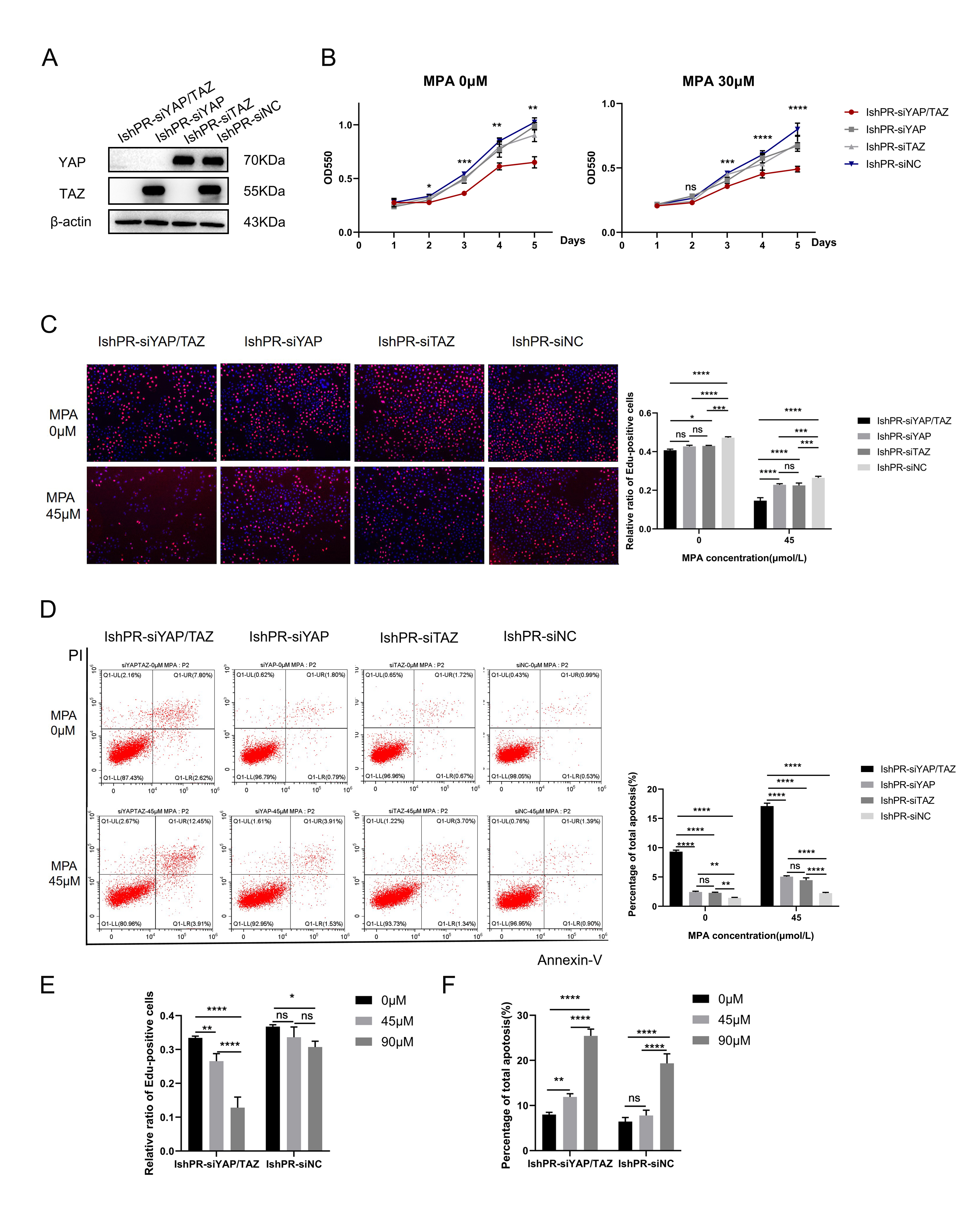

Supplement: Supplementary file 4 — Supplementary Figure 2 [file 41420_2023_1319_MOESM4_ESM.jpg]

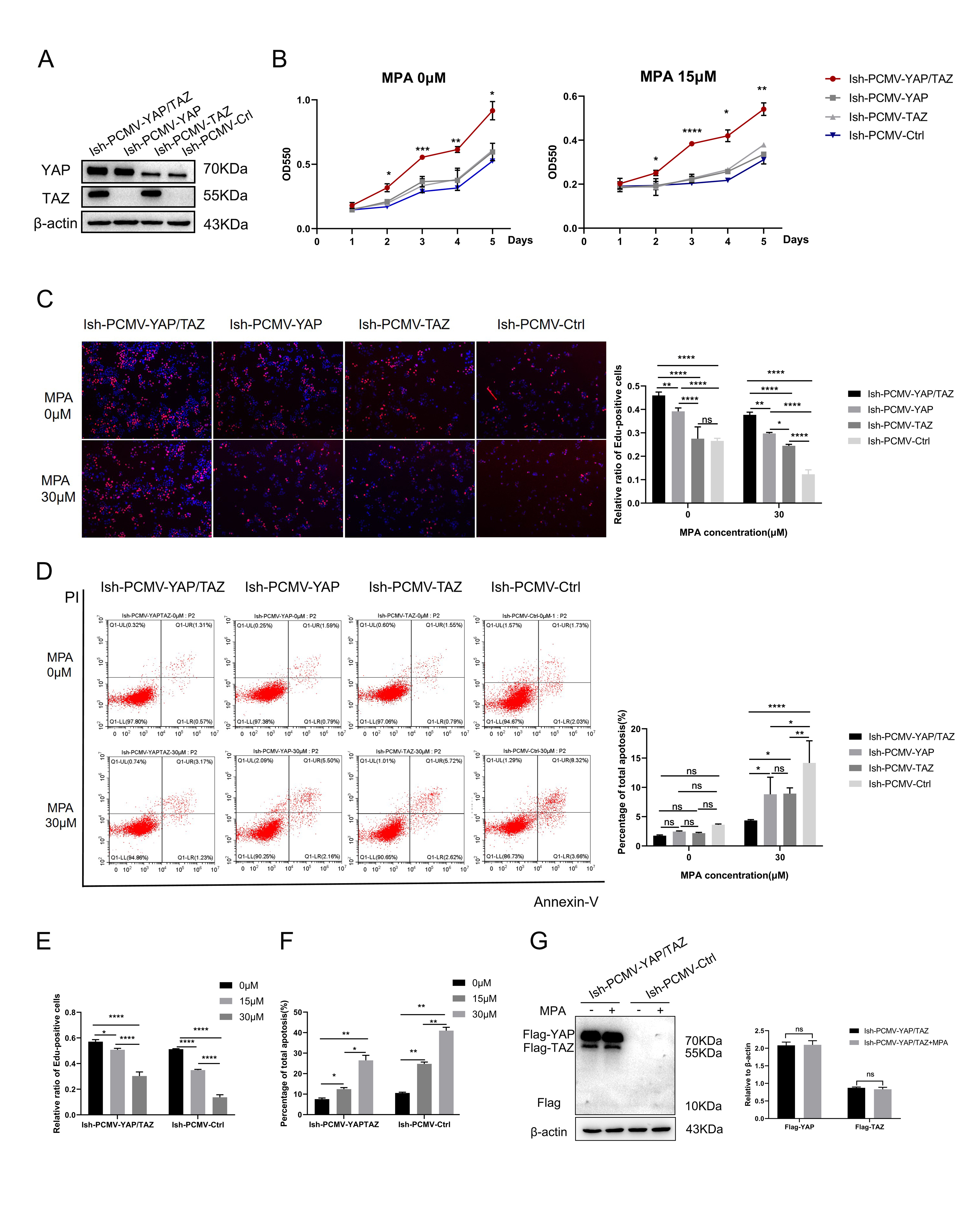

Supplement: Supplementary file 5 — Supplementary Figure 3 [file 41420_2023_1319_MOESM5_ESM.jpg]

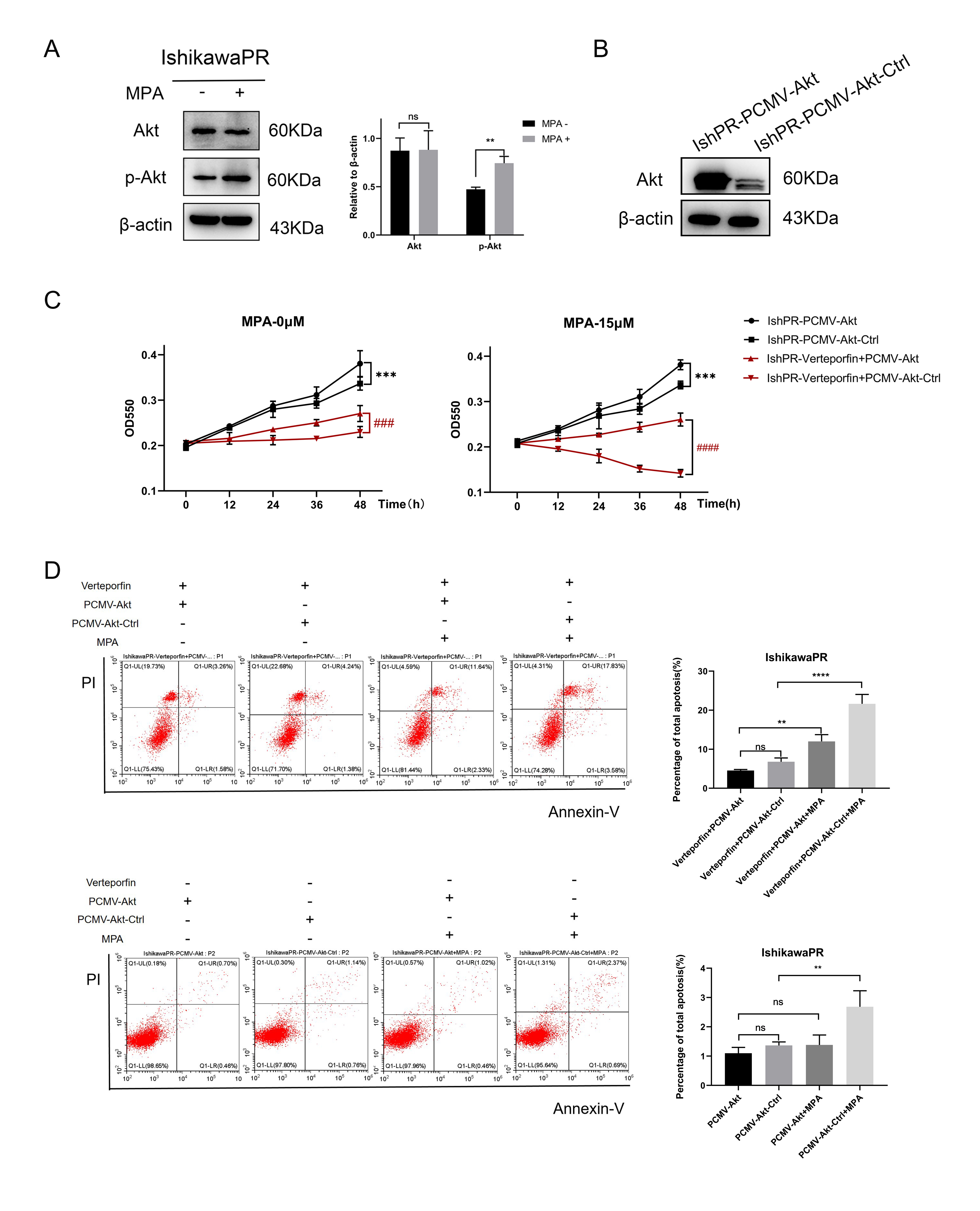

Supplement: Supplementary file 6 — Supplementary Figure 4 [file 41420_2023_1319_MOESM6_ESM.jpg]

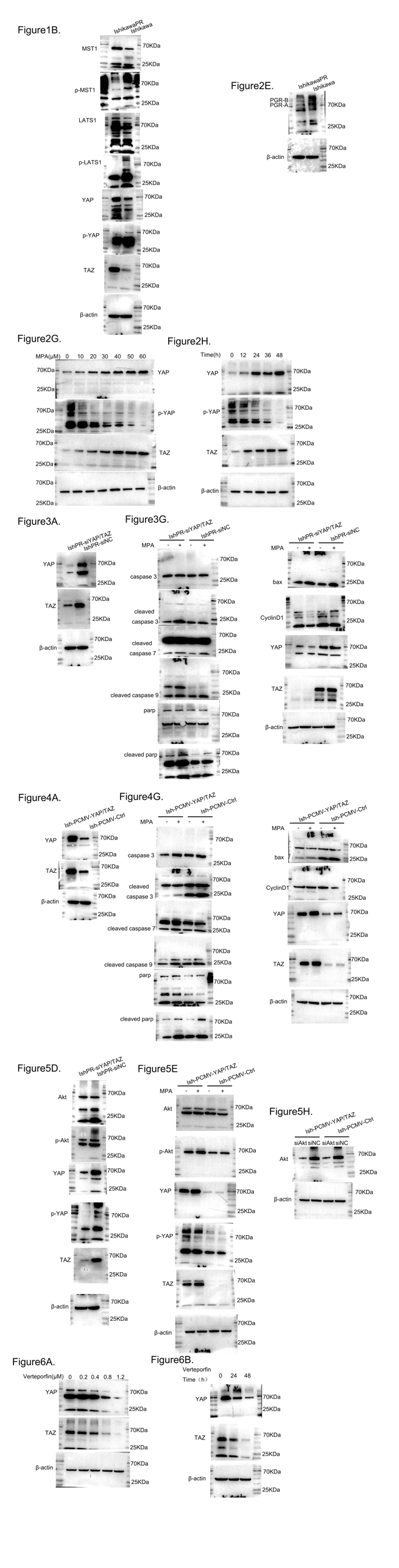

Supplement: Supplementary file 9 — Original Data File [file 41420_2023_1319_MOESM9_ESM.jpg]

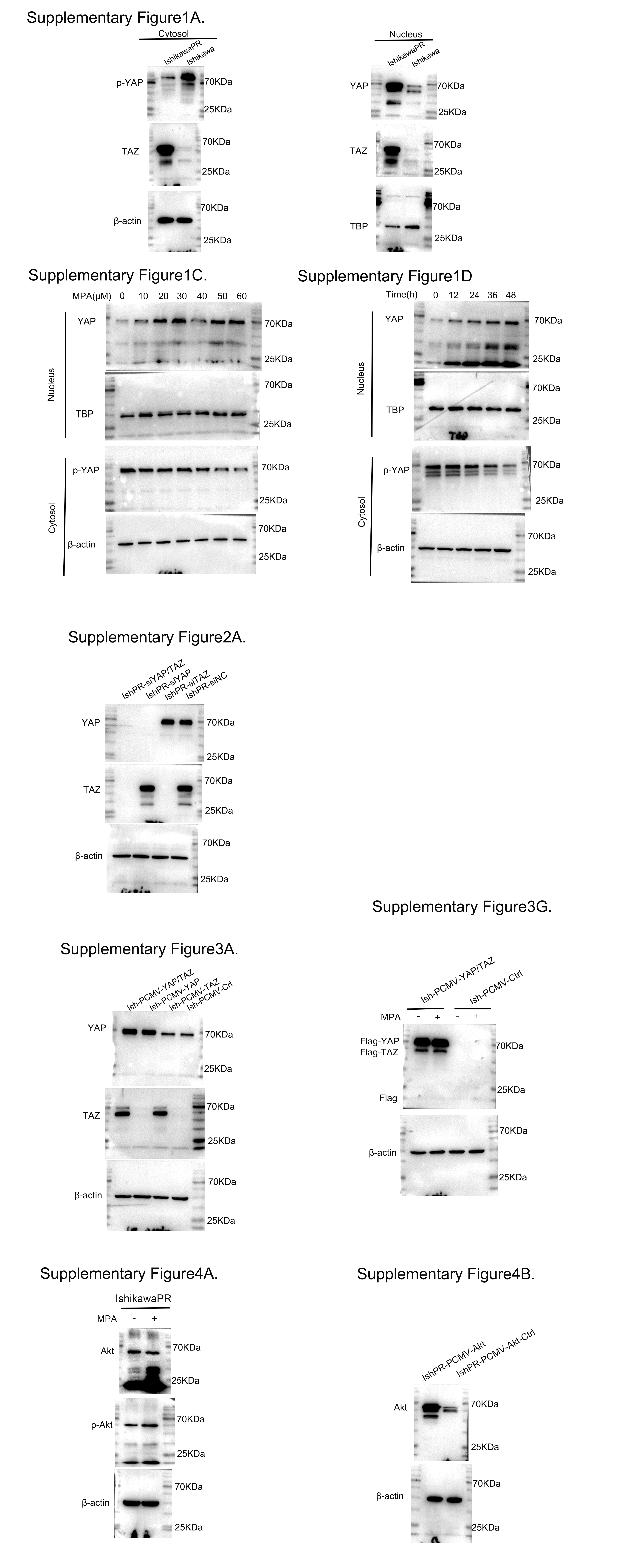

Supplement: Supplementary file 10 — Original Data File [file 41420_2023_1319_MOESM10_ESM.jpg]
