# Supplementary material for: Verteporfin reverses progestin resistance through YAP/TAZ-PI3K-Akt pathway in endometrial carcinoma
Source: Cell Death Discov. 2023 Jan 25;9:30. doi: 10.1038/s41420-023-01319-y (PMC9873621; doi:10.1038/s41420-023-01319-y)
Supplement: Supplementary file 7 — Supplementary Tables [file 41420_2023_1319_MOESM7_ESM.docx]

**Supplementary Table 1**. CI value of inhibitory effect of MPA combined with Verteporfin in IshikawaPR cells.

| MPA(μM) | Verteporfin(μM) | CI Value |
| --- | --- | --- |
| 30 | 0.2 | 0.881 |
| 45 | 0.3 | 0.783 |
| 60 | 0.4 | 0.772 |
| 90 | 0.6 | 0.746 |
| 120 | 0.8 | 0.700 |

**Supplementary Table 2**. The information of primers used in this study.

| **Gene name** | **Primer sequence** | |
| --- | --- | --- |
| YAP | Forward | TCCCTCGAACCCCAGATGAC |
|  | Reverse | TGTCCCAGGAATGGCTTCAA |
| TAZ | Forward | GAATCCGAAGCCTAGCTCGT |
|  | Reverse | CGAGTGCGAGCGGACAT |
| PGR | Forward | TGGAAGGGCAGCACAACTAC |
|  | Reverse | TGACAGCACTTTCTAAGGCGA |
| β-actin | Forward | GAAGAGCTACGAGCTGCCTGA |
|  | Reverse | CAGACAGCACTGTGTTGGCG |

**Supplementary Table 3**. The information of targeting sequence of RNAi used in this study.

| **Gene name** | **Targeting sequence** |
| --- | --- |
| siYAP | 5’CUGCCACCAAGCUAGAUAATT3’ |
| siTAZ | 5’CCUGCCGGAGUCUUUCUUUTT3’ |
| siNC | 5’UUCUCCGAACGUGUCACGUTT3’ |

**Supplementary Table 4**. The vector constructs of overexpression YAP and TAZ.

| **Gene name** | **Vector construct** | **Vector** |
| --- | --- | --- |
| PCMV-YAP | Ubi-MCS-3FLAG-SV40-EGFP-IRES-puromycin | GV358 |
| PCMV-TAZ | Ubi-MCS-3FLAG-CBh-gcGFP-IRES-puromycin | GV492 |
